# Exploring Empathic Communication Among Community Health Workers: Applying the ENACT Tool in Two South African Sites

**DOI:** 10.1177/15248399241285888

**Published:** 2024-10-05

**Authors:** Christina A. Laurenzi, Stephan Rabie, Sihle Mamutse, Sarah Skeen, Nicola Jansen van Vuuren, Rosanne Neethling, Sally Field, Simone Honikman

**Affiliations:** 1 Institute for Life Course Health Research, Department of Global Health, Stellenbosch University, Tygerberg, South Africa; 2 University of Cape Town, Cape Town, South Africa; 3 University of Amsterdam, Amsterdam, The Netherlands

**Keywords:** empathic communication, community health workers, quality improvement intervention, client-provider communication, ENACT rating tool, health care interactions, South Africa

## Abstract

*Introduction*. Effective empathic communication between health care providers and patients is an essential part of health care. In resource-poor contexts, evidence is needed to understand the quality and content of health care communication within real-life clinical engagements. We used the existing Enhancing Assessment of Common Therapeutic Factors (ENACT) tool to measure empathic communication skills among a group of community health workers (CHWs) receiving a novel quality improvement intervention called Nyamekela4Care in South Africa. *Methods*. In two resource-limited sites in the Western Cape, South Africa, we audio-recorded CHWs, with consent, in routine client consultations at baseline and postintervention. All sessions were in Afrikaans. We used the adapted ENACT tool to rate recordings at both timepoints, assessing 11 items including communication skills, emotional engagement, process and interaction. We used ANOVA to assess preimplementation and postimplementation differences in empathic communication, and analyzed coders’ feedback on the coding process itself. *Results*. We analyzed *n* = 66 recordings from 11 CHWs, observing positive directionality overall, with most skills improving over time. Despite near-significant improvements in communication delivery (*p* = .083), self-confidence/groundedness (*p* = .029) significantly changed but in the opposite direction. Large effect sizes were observed in verbal communication, responsiveness to client, and identifying external resources, with no significant difference between timepoints. ENACT was feasible to apply to audio recordings; inter-coder reliability was suboptimal despite coder training and ongoing monitoring and support. *Discussion*. Quality improvement interventions may improve empathic skills in diverse contexts, and our results demonstrate how empathic skills could be more routinely assessed in low-resource health care settings.

Effective empathic communication between health care providers and the individuals they serve is essential to supporting better patient health outcomes (Moudatsou et al., 2020). Empathic communication is rooted in a broader concept of care that draws on empathic practices. This care may involve multidimensional cognitive, affective, and behavioral aspects of how providers interact with patients—with practices including effective communication as well as providers’ ability to understand and express this understanding to patients (Wilkinson et al., 2017; World Health Organization, 2018). According to the World Health Organization (WHO), provider empathy is critical in fostering relationships with patients and promoting shared decision-making that values patients’ rights and perspectives (World Health Organization, 2015). Provider empathy may facilitate positive outcomes for providers themselves, such as reducing errors by improving their ability to detect patients’ experiences and concerns. This practice can lessen health care provider stress and burnout, improving workplace culture and shortening the emotional distance between providers and patients (Kerasidou et al., 2021), which can lead to improvements in care provision and health outcomes. Similarly, empathic communication can also support improvements in patient outcomes along both physical and psychological dimensions (Howick et al., 2018).

However, providers working in settings with limited resources and high demand for services often face numerous, competing demands—including long working hours, understaffing, limited time with patients, and high demand to meet operational targets (Kerasidou et al., 2021). These factors may disrupt providers’ ability to prioritize empathic communication in care (Perry et al., 2014). The interpersonal requirements of everyday patient care can lead to provider burnout and depersonalization, straining their ability to provide quality patient care (Blacklock et al., 2016; Nepal et al., 2020). There are also foundational gaps in provider training on empathic approaches to care (Bonvicini et al., 2009). Often, practicing empathic communication in over-burdened settings becomes less of a priority to both health care providers and their managers than trying to provide care for as many patients as possible. However, importantly, expanding the use of empathic communication in these settings may lead to higher-quality health interactions, decreasing health service utilization and lessening demand on health systems (Howick et al., 2018).

Many of these challenges are also present for other cadres in the health system, including community health workers (CHWs). Community-based approaches to service delivery have been positioned as a way to reduce strain on health systems, and CHWs may be recruited to address both preventive and promotive health challenges in the same communities in which they themselves live (Laurenzi, Skeen, Coetzee, et al., 2021; Lehmann & Sanders, 2007). While CHWs may not be facility-based, they are often given a wide range of responsibilities to reduce strain on health systems (task-sharing) (Limbani et al., 2019), and increasingly taking on additional tasks in a process sometimes called “task-dumping” (Jacobs et al., 2021). CHWs may similarly struggle to use empathic communication with their clients because of the nature of their relationship, which may be more informal or peer-like than those between patients and facility-based health care providers, as well as more limited training and support in their roles (Laurenzi, Skeen, Rabie, et al., 2021).

Contextual factors also overlap with these system-specific demands. In South Africa, the public health care system is fragmented and inequitable, in large part due to a historical legacy of dispossession and discrimination prior to and during apartheid (Maphumulo & Bhengu, 2019). There is poor alignment between policy and implementation, and patient-provider hierarchies are pronounced. Multiple crises, including the HIV epidemic as well as chronic leadership failures, have increased the intractability of health system changes.

Existing research on health service quality focuses predominantly on patient experiences in understanding how empathic communication occurs (Al-Habbal & Arawi, 2020; Elayyan et al., 2018; Nepal et al., 2020). To respond to a gap in evidence from health care providers—especially in low-resource settings where it is critical to improve quality of care—measures are needed to identify and characterize empathic communication. The observational Enhancing Assessment of Common Therapeutic Factors (ENACT) rating scale was developed to measure these skills in lay providers specifically. It has been used in low- and middle-income countries (LMICs) including Ethiopia, Jordan, Lebanon, Nepal, Pakistan, and South Africa (Ng et al., 2021), and to assess provider competence in psychological care (Asher et al., 2021), disaster response (Jordans et al., 2020), and mental health stigma reduction (Kohrt et al., 2018). Overall, the tool has been used to identify gaps in a range of foundational provider skills in health-related communication and show improvements over time (Jordans et al., 2021), including for community non-specialists.

However, while ENACT has been found to be useful in clinical observational settings, its broader applicability across cadres has not been tested. More specifically, there has been limited investigation into interventions to enhance skills and communication among CHWs in these settings. We aimed to test the use of ENACT to measure empathic communication skills among a group of CHWs, who were receiving a novel quality improvement intervention called Nyamekela4Care (N4C) (Rabie et al., 2022), in two sites in South Africa.

## Methods

This study is a substudy of a larger investigation of the acceptability, feasibility, and preliminary effectiveness of the N4C intervention (Rabie et al., 2022). N4C, specifically designed for implementation in low-resource settings, leverages peer relationships to engage care service providers (including those involved in health care and social services) in training and support sessions that are integrated into routine meeting spaces. N4C provides a 10-session manualized, structured format to enhance job-related training, empathic skills development, case sharing, work administration and self-care practices. The 10-section empathic engagement component includes accessible theory on counseling skills and practice exercises, divided to be covered in each meeting. The skill set selected for the empathic engagement component was aligned with the skills assessed in the ENACT tool. Our study was informed by the Caring Communication Theory as the theoretical framework to investigate the empathic communication skills within the broader context of care. Rooted in the works of Watson & Foster and Swanson, Caring Communication Theory underscores the moral and ethical imperative of caring in health care communication (Swanson, 1993; Watson & Foster, 2003). The theory posits that effective care involves not only technical competence but also a genuine expression of concern and compassion.

## Setting and Sample

This study was implemented in two communities in the Western Cape province of South Africa—one rural (Site 1) and one peri-urban (Site 2) community, both of which have a majority low-income population. While health systems challenges linked to provider empathy exist across diverse contexts in LMICs, they are particularly pronounced in South Africa. Repeated efforts to bridge gaps in accessibility and affordability have been unsuccessful, and health outcomes remain poor (Burger & Christian, 2020; Maphumulo & Bhengu, 2019). In a recent global comparison of 48 countries, South Africa was ranked in the bottom eight in terms of quality care in public health services (Burger & Christian, 2020).

Both study communities were pilot learning sites for the Western Cape Government’s Community-Orientated Primary Care (COPC) initiative, which promotes intersectoral collaboration to strengthen health systems and promote health service access (Mash et al., 2019). Participants who took part in the N4C program included primary care facility staff members (nurses and doctors) and CHWs from two partner non-profit organizations that collaborate with the respective clinics. More details on the development of the N4C curriculum can be found elsewhere (Rabie et al., 2022).

## Data Collection Procedure

To collect information on empathic communication skills in practice, we approached all providers enrolled in the N4C program evaluation and obtained informed consent from 18 health care providers to audio-record their consultations with clients. Providers were notified that they were being recorded to measure how they interacted with their clients, but researchers did not specifically mention assessing empathic engagement.

At baseline, a sample of consultations (up to four per provider) were audio-recorded. The consultations selected for recording comprised the clients seen on a given day, when both the researcher (S.R.) and the provider were available. The researcher visited facilities and/or conducted home visits to recruit and obtain informed consent from clients prior to their consultations. Clients were approached in private, and the purpose of the audio recording was explained. Once informed consent was obtained, the recording device was placed unobtrusively in the room in which the consultation was to take place, and providers were asked to proceed with consultations as usual. The researcher was not present during the consultations but was available to discuss any questions or concerns before or after the sessions. For the follow-up assessment, the same sample of providers was revisited to audio-record consultations. Although the same procedures described above were followed, a new sample of clients was engaged to integrate the study into the daily routines of providers, similar to the process used by the lead author in a prior study (Laurenzi et al., 2020).

Between November 2018 and December 2019, 66 audio recordings were taken at two timepoints, baseline (pre-N4C implementation) and endline (2–3 weeks postimplementation) from participating providers. All sessions conducted were in Afrikaans, the preferred language of both the providers and their clients.

Ultimately, only data from CHWs were used, due in part to logistics as well as their representation among N4C participants. For clarity, we will refer to participants from this point as CHWs.

## Applying the ENACT Rating Scale

The Enhancing Assessment of Common Therapeutic Factors (ENACT) rating scale was developed by Kohrt and colleagues in 2015 in Nepal. ENACT was designed to be used to enable peer and supervisor assessment of competence of non-specialist mental health care workers participating in task-sharing initiatives for psychosocial support. The measure was adapted and validated for South Africa by the Center for Public Mental Health; we utilized the adapted version (ENACT-SA) to score the empathic communication skills of CHWs (Spedding et al., 2022). Domains included communication skills; emotional engagement (responsiveness to and rapport-building with client); process and interaction (managing confidentiality and linking to additional resources); and other counselor qualities (appropriate self-confidence and sensitivity to race, socioeconomic status, and other factors linked to client context). ENACT-SA consists of 12 items coded on a Likert-type scale with four response options: 0 (*Not assessed*), 1 (*Needs improvement*), 2 (*Done partially*), and 3 (*Done well*) (see Supplemental File 1 for the full tool).

The N4C intervention includes a prominent component on empathic engagement and basic counseling skills that included information and practice exercises. The set of skills selected was loosely aligned with the skills domains in the ENACT-SA tool.

## Scoring Procedure

Two masters-level Afrikaans-speaking research assistants were trained by the lead author on how to apply the ENACT-SA tool, and were responsible for scoring all audio recordings. Initial independent coding took place in parallel, with coders completing scoring of the same audio and convening biweekly to discuss discrepancies and rationale. After an initial set of *n* = 9 audio recordings were cross-coded and checked for intercoder reliability, each coder proceeded with a separate list of audio recordings. A total of *n* = 16 audio recordings were cross coded by both research assistants. During the coding process, we decided to omit one item (Item 10; Flexibility: ability to adapt to client) due to lack of requisite information to score this item.

Prior to coding, eleven recordings were excluded, four from baseline and seven from endline data collection. Audios were omitted from scoring for poor audio quality, as well as in select cases where recordings were less than 2 minutes long, where the topic of conversation was purely informational and not focused on health behaviors or individual challenges, and/or in cases where there were more than three missing values in the scoring. All audios were scored in their original language, Afrikaans.

## Data Analysis

ENACT-SA scores from both coders, provided as individual entries for each audio identifier, were collated into an Excel template iteratively. Double-coded audio recordings were entered side-by-side to enable intercoder reliability checks. In addition to scores, examples of each coder’s rationale for their score per item were extracted for closer review. An average score per item, per service provider, was calculated. Where both coders scored one audio recording, two scores were used to calculate the average.

Data were analyzed by one of the study authors (S.R.) using SPSS 27.0. We utilized a within-group, two-way analysis of variance (ANOVA) to assess the differences preimplementation and postimplementation of N4C on empathic skills. We assessed significance at alpha levels of 0.05.

## Ethics

This study obtained ethical permission from the University of Cape Town’s Human Research Ethics Committee (HREC 144/2016). Participation in this study was completely voluntary, and all participants provided informed consent at each data point. Participants were ensured that all data was anonymized and kept confidential. Participants were reassured that they were free to withdraw from the study during any time of the study, and that their employers would not have access to any data shared with the research team.

## Results

We describe results from this work divided into (a) scores across ENACT domains and items, and (b) application of the ENACT tool.

## Scores Across ENACT Domains and Items

The final analytic sample consisted of audio recordings from 11 providers (all of whom were CHWs), roughly evenly split across the peri-urban site (*n* = 5) and the rural site (*n* = 6) (Table 1). At baseline, between 2 and 4 sessions were recorded per provider; at endline, between 1 and 4 sessions were recorded. To our knowledge, no clients were repeatedly recorded between baseline and endline, the unit of focus being the CHW. The majority of visits were home-based; only one CHW (Participant 8) was based at a primary health care facility, where their consultations were recorded.

**Table 1:** Recordings by Health Care Provider and Site

*Participant*	*Baseline recordings*	*Endline recordings*	*Site*	*Employment type*	*Age*	*Education level*	*Years of service*
1	3	4	Rural	Nonprofit organization (NPO)	55	Some high school	8 years
2	3	3	Rural	NPO	49	Completed high school	3 years
3	2	3	Rural	NPO	47	Some high school	13 years
4	3	3	Rural	NPO	52	Some high school	2 years
5	1	2	Rural	NPO	22	Postgraduate diploma in HIV care	3 months
6	4	1	Peri-urban	NPO	26	Completed high school	6 years
7	3	1	Peri-urban	NPO	23	Completed high school	2 years
8	3	1	Rural	DoH facility	54	Some high school	28 years
9	3	3	Peri-urban	NPO	53	Some high school	6 years
10	2	1	Peri-urban	NPO	21	Some high school	2 years
11	3	3	Peri-urban	NPO	45	Completed high school	4 years

Average scores per provider across each scored empathic skills item and across both time points are reported in Table 2.

**Table 2:** Scores From ENACT

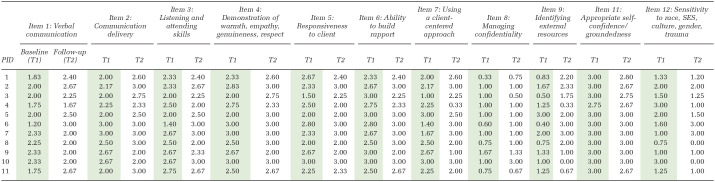																						

Table 3 shows results from the analysis comparing empathic communication skills scores at baseline and follow-up (Table 3). Overall, we identified positive directionality, with most skills improving over time. There was a notable improvement in participants’ baseline and follow-up in communication delivery (*p* = .083) which includes considerations of vocal tone and volume. However, the only finding significant at *p* = .05 level was a worsening of participants’ baseline and follow-up in self-confidence/groundedness (0.029) which includes the degree to which the provider is relaxed, calm, comfortable and appropriately confident.

**Table 3:** Analysis of Within-Subjects ANOVA

*Items*	*Baseline (mean, SD)*	*Follow-up (mean, SD)*	*F*	*p value*	*Effect size with partial eta squared*
Item 1: Verbal communication	1.82 (0.51)	2.10 (0.80)	1.90	.198	0.160
Item 2: Communication delivery	2.46 (0.39)	2.74 (0.35)	3.712	.083	0.271
Item 3: Listening and attending skills	2.21 (0.57)	2.17 (0.85)	0.041	.844	0.004
Item 4: Demonstration of warmth, empathy, genuineness, respect	2.64 (0.33)	2.62 (0.50)	0.012	.916	0.001
Item 5: Responsiveness to client	2.15 (0.85)	2.41 (0.53)	1.850	.204	0.156
Item 6: Ability to build rapport	2.46 (0.77)	2.38 (0.86)	0.222	.648	0.022
Item 7: Using a client-centered approach	1.95 (0.64)	1.88 (1.06)	0.037	.852	0.004
Item 8: Managing confidentiality	0.84 (0.38)	1.02 (0.74)	0.761	.404	0.071
Item 9: Identifying external resources	0.98 (0.59)	1.39 (1.03)	1.376	.268	0.121
Item 11: Appropriate self-confidence/grounded-ness	2.98 (0.08)	2.87 (0.16)	6.521	.029*	0.395
Item 12: Sensitivity to race, socioeconomic status, and culture	1.48 (0.92)	1.22 (1.08)	0.390	.546	0.037

* *p* < .05.

Large effect sizes were observed in empathic skills including verbal communication, responsiveness to client, and identifying external resources, despite no significant difference between baseline and follow-up. Empathic skills in managing confidentiality showed a medium/moderate effect size, while small effect sizes were recorded for listening and attending skills, demonstration of warmth, empathy, genuineness, respect; ability to build rapport; using a client-centered approach; and sensitivity to race, socioeconomic status, and culture.

## Application of the ENACT-SA Tool

Overall, ENACT-SA was found to be feasible to use and to apply to audio recordings, and coders were given opportunities for inception training, ongoing monitoring and support, and routine check-in calls to discuss discrepancies and rectify divergent interpretations of how items should be coded.

Inter-coder reliability was moderate to low, with an overall agreement rating of 65.5%. Items related to counselor qualities had the highest rates of inter-coder agreement (73.3%) whereas items related to communication had low agreement (55.6%). There was moderate agreement in the emotional engagement domain (68.3%) and the process/interaction domain (66.7%). In addition, while initially coded audio recordings had higher levels of agreement, audio recordings that were coded after more time had elapsed since training were found to be less reliable. Audio recordings with poorer audio quality had higher rates of disagreement.

Table 4 shows examples of how both coders applied criteria for each item, with qualitative descriptions of each item and scoring option.

**Table 4:** Descriptions of Coding Rationale Per Item and Score

*Items*	*1*	*2*	*3*
Item 1: Verbal communication	CHW initially does not use the opportunities that the client presents. The CHW could benefit from training on how to facilitate deeper conversation and being interested in the client’s stories. The CHW places too much focus on telling the client what she is doing (during the wash session) and is not in tune with the conversation and possible emotional needs.	The CHW gave the client a lot of opportunity to talk and express herself. The CHW could work on reflecting and summarizing.	Clarifies what client says by repeating as a question, to ensure understanding. Asks open-ended questions allowing client to elaborate, and carer continues the conversation with follow-up questions, ultimately discussing topics in detail.
Item 2: Communication delivery	CHW is not good at keeping the conversation flowing and exploring topics further.	Vocal tone and volume is good, but no real attempts to show interest and to facilitate a conversation.	The CHW talks to the client throughout the session and talks her through the procedure. The client is not able to respond verbally, but the CHW has a soft and appropriate vocal tone and volume.
Item 3: Listening and attending skills	The CHW does not always finish listening to the family member of the client (a child) and sometimes cuts her off. She also calls someone (with regard to the client’s treatment) while the client’s family member is still talking.	For the most part the CHW shows good listening skills and is attuned to the client. There was one instance at the beginning of the session where the client spoke about a particular day she had pain, and the CHW did not follow-up on that, but instead asked if the client is happy with their services.	CHW does follow client’s story, and asks follow-up questions or makes appropriate comments. When CHW can’t hear what client is saying, she does continue asking until she can hear.
Item 4: Demonstration of warmth, empathy, genuineness, respect	CHW is generally warm and respectful. There is an occasion where the CHW comes across as a bit cross (raises voice).	The CHW is warm and treats the client with respect but does not always show appropriate empathy.	Even when the client is being difficult and wanting to resist being washed, the CHW is warm and shows empathy and tries to distract the client.
Item 5: Responsiveness to client	Praise was given once, but for the most part it felt like the CHW did not acknowledge what the client said and does not give constructive feedback.	Could work on praising the client for small things. The CHW acknowledged the client.	In this session the CHW was very responsive to the client’s physical state and talked her through the whole wash. She praised the client for how she always helps to lift her arms.
Item 6: Ability to build rapport	CHW does ask some questions about client follow-up but refrains from asking again along the interaction or throughout the visit.	CHW is focused on client and creates an environment in which the client is prioritized and attended to. CHW builds some conversation about everyday things that creates a comfortable environment, but there are long periods of silence. Topics are not always further elaborated on or explored.	They seem to have a good relationship/understanding, and the CHW seems to be comfortable with the whole household. Although it is not always a very warm relationship, it seems to be in tune with the client’s personality/norm. The client’s agenda is definitely prioritized, and she seems to be very comfortable with the CHW.
Item 7: Using a client-centered approach	The CHW says the same thing over and over and does not check in to assess the client’s understanding.	The CHW did some checking in to ensure understanding but does not leave a lot of opportunity for the client to ask questions.	The CHW listened to the client and worked well with her to address her needs. The client also had the space to ask questions.
Item 8: Managing confidentiality	Confidentiality was not discussed, and the setting did not seem conducive to possibly having a confidential conversation.	CHW does occasionally address confidentiality	CHW does mention that the recording is only meant for the ears of the researcher, not for the CHW network or others at [institution].
Item 9: Identifying external resources	The need for resources was not discussed. At one stage the client mentioned that she is alone at home a lot with no one to talk to. It did not seem to be a big problem and was mostly said in a joking way, but the CHW could have followed up on this and try to discuss a solution for this.	Tells the client that she will make an appointment for her at the clinic for her blood pressure but misses the opportunity to refer to psychological services.	The CHW plans on bringing a dietician to the client’s home and tells him to ask the doctor about his pain medication. She also gives him tips on how to exercise and manage his pain.
Item 11: Appropriate self-confidence/groundedness	The CHW does not seem confident in her work or know what to do even when the client does not want to comply.	The CHW seems confident in her abilities and does not doubt herself. She did come across as being rushed, however.	The CHW seems confident in her work and knows what to do even when the client does not want to follow advice.
Item 12: Sensitivity to race, SES, culture, gender, trauma	CHW does not seem sensitive to how the client’s psychological disorder impacts on her emotions and life.	CHW shows little sensitivity to client’s health issues.	The CHW is sensitive/caring about the fact that the patient has two amputated legs. The trauma of what led to the amputation was not discussed, but the CHW was never harsh or insensitive.

## Discussion

Our analysis draws out insights about both the potential supportive role of an intervention to improve empathic skills, as well as about how these skills may be measured in diverse contexts. Our pre–post examination of empathic skills, using audio recordings of provider-patient engagements, garnered limited significant findings, but drawing from a small sample, provided some promising results on directionality. While not able to be conclusive, these data on directionality and effect show some sensitivity to change over time and are illustrative for considering the practical application of the ENACT tool. Scores in verbal communication, communication delivery, and responsiveness to client all improved at the post-N4C intervention time point. Prior studies have shown how highly patients value responsiveness in communication from providers (Al-Habbal & Arawi, 2020), highlighting how important these aspects of clinical communication are in enhancing overall quality care. Importantly, understanding and identifying ways to strengthen these types of skills is critical for CHWs and their supervisors, as this cadre is at the forefront of health promotion and prevention efforts in many global settings (Jeet et al., 2017).

Scores in appropriate self-confidence/groundedness emerged as significant between time points, however, not in the anticipated direction. This item emphasizes the importance of nurtured relationships between providers and patient, through assurances and providing adequate information throughout the care interaction (Spedding et al., 2022). While both time points showed very high scores overall, lower scores in self-confidence may be expected in the aftermath of an intervention, as providers may question their typical way of interacting and struggle to internalize and apply new knowledge. However, N4C aims to enhance self-confidence; the lower endline score may be attributed to factors external to the N4C intervention itself. Our previous feasibility paper of N4C in these sites outlines contextual considerations at the time of data collection, such as uncertainty of employment, that may have shaped this finding (Rabie et al., 2022).

Certain skills may be more ingrained in how CHWs already carry out their work—for instance, demonstration of warmth, empathy, genuineness and respect scored relatively highly across both time points, in line with other findings illuminating these qualities in CHWs (Laurenzi et al., 2020). Similarly, with more limited shifts for some items such as listening and attending skills or using a client-centered approach, alternative training and support may be required to see more substantial, sustained changes. Possible preexisting relationships with clients may influence how readily a CHW may use directive statements or respect the client’s autonomy to make their own health decisions. Other areas scored lower overall, including identifying external resources and managing confidentiality, although there was a larger effect size observed for the former at follow-up—indicating that the N4C intervention training may be able to support improvements in CHW competencies or how they enable clients to take up opportunities for support. To be optimally effective, empathic communication skills training programs must consider CHWs’ existing capacities, and the roles CHWs are expected to fulfill (Scott et al., 2018).

### Reflections on the Coding Process and Broader Applicability of the ENACT-SA Tool

Beyond considering the shifts in observed skills across the CHW sample, our analysis highlights the applicability of the adapted ENACT-SA tool for contexts of community-based service delivery. Our process was strengthened by a robust training process and opportunities for inter-coder engagement; however, we also encountered limitations to both the process and the tool’s application.

To test the application of the ENACT-SA tool, our team made certain decisions to reduce costs and consider practical value. ENACT-SA was applied to previously-captured audio recordings coded in vivo by research assistants who spoke the same language as the CHWs and clients, rather than applying codes to transcripts. While other coded analyses of CHW visits have relied on translated transcripts (Laurenzi et al., 2020), recent studies have also utilized audio recordings to assess provider competencies via the ENACT tool (Mwenge et al., 2022). The decision to code audio recordings directly reduced costs and personnel required, and enabled coders to capture nuances in audio recordings not readily captured in transcripts.

Inter-coder reliability was lower than ideal, posing some important questions for future applications of ENACT-SA. Because of the frequency of discussion and deliberation surrounding double-coded recordings, we identified several reasons for more persistent inter-coder disagreement. Audio quality was one challenge; certain household environments were busier and less conducive to high-quality audio recordings. We also identified differing interpretations between coders about how to distinguish between codes based on similar constructs—for instance, when to code something as falling under “verbal communication” versus “communication delivery.” The rubric for these items appeared sufficiently distinct, but in practice, these two items frequently differed between coders. Generally, certain items were more straightforward than others, and able to be reliably double-coded. Beyond the limits posed by the tool, it may be that some types of CHW-client interactions are harder to find consensus about than others. At times, the coders identified limitations within the tool itself. Certain items within the scale were less relevant to settings where CHW deliver care. In some cases, this was specifically linked to utterances by the CHW. For example, Item 6 (ability to build rapport) contained the scoring consideration “counsellor introduces him/herself,” and Item 8 (managing confidentiality) aimed to capture assurances of confidentiality within the clinical interaction. Both of these provisions, however, are less common in routine CHW-client interactions, as CHWs typically build long-term relationships with clients and often conduct monthly or bi-monthly visits, depending on clients’ needs and programmatic expectations (Murphy et al., 2021; Thomas et al., 2021). Similarly, in cases where established relationships between clients and CHWs existed, Item 9 (identifying external resources) presented coding challenges, as it was difficult to determine whether available resources had previously been discussed. Coders aimed to balance between being flexible across recordings to give CHWs leeway, and applying the same standards across all recordings.

Other practices were identified as being inconsistently applied, or unevenly distributed, across the duration of CHW-client interactions. For instance, in certain recordings, CHWs satisfied some criteria under Item 4 (demonstration of warmth, empathy, genuineness, and respect)—coming across as respectful and friendly, but also missing opportunities to show empathy as a client shared a difficult story within the same consultation. Additional training in active listening, or opportunities for open client-provider dialogue, may improve these empathic qualities (Tan et al., 2021).

### Implications for Further Research and Practice

Our analysis and methodological considerations carry several implications for future research and practice. The ENACT tool provides conceptual and structural guidance for training of CHWs and other health care providers, as well as a mechanism to routinely assess their skills in practice. Real-time observation and audio recording review are both avenues for completing such assessments, and may be feasible across a range of resource and contextual constraints.

The ENACT tool has been more recently integrated into the WHO’s EQUIP curricula, as a competency-based training and assessment resource adaptable to the contexts and needs of non-specialist and specialist providers (Kohrt et al., 2020). EQUIP is intended for trainers, supervisors and project managers tasked to implement psychosocial support across cultures and interventions.

The N4C intervention has broad application for integration into clinical and community-based care settings, which has important implications for the potential of CHWs to be agents of health promotion in the communities in which they work. However, the diverging experiences of its implementation across sites, detailed in a prior publication (Rabie et al., 2022), highlight the need to ensure high-quality implementation and consider underlying factors that may complicate quality of care. It is also important that empathic skills are better integrated into clinical education and training through supervision and supportive skills monitoring, with diverse tools to measure these skills (Yu et al., 2022). While most clinical education does emphasize empathic skills training, these approaches differ in their concept of empathy and training methods, content, and duration (Paulus & Meinken, 2022). For CHWs, for instance, training curricula can extend anywhere between a few days (Redick et al., 2014) or 6 weeks (Tomlinson, 2014). For empathic care to be extended to diverse clientele, it is important that these skills are taught to be applied flexibly, across the continuum of care, and that supervision and mentorship focuses on ensuring results in both health outcomes and quality of care (Mhlongo & Lutge, 2019). Supporting these high-quality client-CHW engagements is a cornerstone to more effective health promotion, and essential for broader public health priorities in countries like South Africa (LeBan et al., 2021).

### Limitations

This study has several limitations. Our small sample, uneven number of audio recordings across CHWs and timepoints, and lack of control group means that we were unable to examine the impacts of the N4C intervention on empathic skills more definitively. In addition, the knowledge of being audio-recorded may have influenced usual CHW behavior and made them more self-conscious about their communication with clients. For the first six audio recordings assigned, coders were not blinded to whether the audios were recorded at baseline or endline; however, this was adjusted shortly after coding began, and the coders were blinded for the majority of the coding. Because of the historical context of apartheid in South Africa, which have informed national ethics guidelines, our two communities were relatively homogeneous, meaning that we did not collect data on race or ethnicity. We are confident that this was not a major limitation, as the CHWs and the clients did not differ by racial/ethnic background. Despite these limitations, we feel that the study provided a solid foundation for considering the broader application of an empathic skills-focused tool.

## Conclusion

Our results show postintervention improvements in empathic skills in CHWs and demonstrate the feasibility of utilizing a novel approach to assess empathy in primary care settings. In high-burden care settings, this approach could be pivotal in the supervision and training of non-specialist care providers to provide psychosocial care—which would be crucial in the context of efforts to reduce the health care treatment gap in LMICs. Importantly for broader health promotion efforts, expanding empathic skills training could also significantly improve health-seeking behaviors by clients, and extend meaningful provider-patient relationships, creating positive foundations for healthier societies.
